# Prospective Association between Total and Specific Dietary Polyphenol Intakes and Cardiovascular Disease Risk in the Nutrinet-Santé French Cohort

**DOI:** 10.3390/nu10111587

**Published:** 2018-10-29

**Authors:** Solia Adriouch, Aurélie Lampuré, Anouar Nechba, Julia Baudry, Karen Assmann, Emmanuelle Kesse-Guyot, Serge Hercberg, Augustin Scalbert, Mathilde Touvier, Léopold K. Fezeu

**Affiliations:** 1 Equipe de Recherche en Epidémiologie Nutritionnelle (EREN), Centre de Recherche en Epidémiologie et Statistiques, Université Paris 13, Inserm (U1153), Inra (U1125), Cnam, COMUE Sorbonne Paris Cité, F-93017 Bobigny, France; a.lampure@eren.smbh.univ-paris13.fr (A.L.); a.nechba@gmail.com (A.N.); j.baudry@eren.smbh.univ-paris13.fr (J.B.); k.assmann@eren.smbh.univ-paris13.fr (K.A.); e.kesse@eren.smbh.univ-paris13.fr (E.K.-G.); s.hercberg@eren.smbh.univ-paris13.fr (S.H.); m.touvier@eren.smbh.univ-paris13.fr (M.T.); l.fezeu@eren.smbh.univ-paris13.fr (L.K.F.); 2 Département de Santé Publique, Hôpital Avicenne, F-93017 Bobigny, France; 3 Biomarkers Group, Nutrition and Metabolism Section, International Agency for Research on Cancer (IARC), 69372 Lyon CEDEX 08, France; ScalbertA@iarc.fr

**Keywords:** cardiovascular disease risk, dietary polyphenols intakes, prospective study

## Abstract

Background: Epidemiological and experimental evidence support a protective effect of dietary polyphenols on chronic diseases, but high quality longitudinal data are needed, including details on categories of polyphenols. Our objective was to investigate the prospective association between total and individual classes and subclasses of dietary polyphenols and the risk of major cardiovascular disease in the NutriNet-Santé cohort. Methods: A total of 84,158 participants, who completed at least three 24 h dietary records, were included between May 2009 and June 2017. Individual polyphenols intakes were obtained by matching food consumption data from the 24 h dietary records with the Phenol-Explorer polyphenol composition database. Multivariable Cox proportional hazards models were used to characterize the associations between dietary polyphenols and the incidence of cardiovascular diseases, comparing tertile T3 vs. T1 of classes and subclasses of polyphenols. Results: Over a median of 4.9 years of follow-up, 602 major cardiovascular events were diagnosed. Intakes of anthocyanins, catechins, and flavonols were strongly inversely associated with cardiovascular disease risk (anthocyanins: Hazard Ratio (HR)_for a 1-point increment of 10 mg/day_ = 0.98 (0.96–0.99, *p* = 0.03, HR_T3vs.T1_ = 0.66 (0.52–0.83), *p*_trend_ = 0.0003; catechins: HR_for a 1-point increment of 10 mg/day_ = 0.98 (0.96–0.99), *p* = 0.02, HR_T3vs.T1_ = 0.74 (0.60–0.91), *p*_trend_ = 0.004; flavonols: HR_for a 1-point increment of 10 mg/day_ = 0.94 (0.90–0.99), *p* = 0.02, HR_T3vs.T1_ = 0.75 (0.61–0.94), *p*_trend_ = 0.006). Intakes of dihydrochalcones, proanthocyaninidins, dihydroflavonols, hydroxybenzoic acids, and stilbenes were also associated with a decrease (13%, 19%, 24%, 24%, and 27%, respectively) in cardiovascular disease risk, when comparing tertile T3 to T1. Conclusions: Higher intakes of polyphenols, especially of anthocyanins, catechins, and flavonols, were associated with a statistically significant decreased cardiovascular disease risk.

## 1. Introduction

Cardiovascular diseases (CVDs) account for almost one third of deaths worldwide [1]; diet plays a decisive role among the modifiable environmental factors involved in the aetiology of CVDs [2]. Polyphenols are bioactive phytochemicals present in plant foods and beverages, classified into four major classes according to their chemical structure (flavonoids, phenolic acids, stilbenes, and lignans), each class being further divided into subclasses [3]. A large number of preclinical and clinical studies suggest a protective role of dietary polyphenols against CVDs [4], and several major risk factors of CVDs, such as hypertension, overweight, dyslipidaemia, or diabetes [5]. A better understanding of the molecular mechanisms involved in CVD progression has led to the hypothesis that the combination of various protective agents—including different types of polyphenols—targeting multiple signaling pathways, might be particularly effective in the prevention of CVDs [4]. Cardiovascular protection by polyphenol intakes may encompass various mechanisms [6,7,8], including anti-inflammatory properties [9], an increase in antioxidant capacity [10], and an inhibition of platelet aggregation and antithrombotic properties [11].

In vivo, studies have also shown that polyphenols may improve endothelial and overall vascular health through the formation of vasoprotective factors, such as nitric oxide [4,12,13], and their effects on the regulation of signaling pathways involved in metabolic homeostasis [14].

Translating these experimental findings to prevention measures for the general population is, however, still limited because of a too limited number of studies focused on primary prevention. These studies have often included a too limited number of individual polyphenols [15], which is far from representing the large diversity of these compounds found in the diet. The Phenol-Explorer database includes food composition data on 502 different polyphenols from 452 different foods [16]. This database has been used in a few longitudinal studies to assess the association between polyphenols intakes and CVDs or mortality risk [17,18,19,20,21]. However, currently available results are somewhat inconsistent with respect to the role of specific classes and subclasses of polyphenols in CVDs. 

Therefore, the aim of the present prospective study was to assess the association between total polyphenol intakes, as well as classes and subclasses of polyphenols, and the risk of incident CVDs, coronary heart diseases (CHD), and cerebrovascular diseases (CD) in the French NutriNet-Santé cohort.

## 2. Methods

### 2.1. Study Population

Participants were volunteers from the NutriNet-Santé study, a prospective observational cohort study launched in May 2009 to evaluate the determinants of eating behaviours, and the relationships between nutrition and chronic disease risk. The NutriNet-Santé study’s objectives and methods have been extensively described in detail elsewhere [22]. Electronic informed consent was obtained from all participants. All procedures were approved by the International Research Board of the French Institute for Health and Medical Research (IRB Inserm no. 0000388FWA00005831) and the French National Information and Citizen Freedom Commission “CNIL” (no. 908450 and 909216). NutriNet-Santé is registered at clinicaltrials.gov as NCT03335644.

### 2.2. Cases Ascertainment

Participants self-declared health events through the yearly health status questionnaire, through a specific check-up questionnaire for health events or at any time through a dedicated interface on the study website. Following this declaration, participants were invited to send their medical records. Then, medical data were reviewed for the validation of major health events. Vital status and causes of death were identified via the national death registry (CepiDC Inserm). The present study focused on all first incident stroke, myocardial infarctions, acute coronary syndromes, and angioplasty diagnosed between the inclusion and June 2017. 

### 2.3. Data Collection

#### 2.3.1. Sociodemographic, Lifestyle, and Anthropometric Data 

Baseline validated [23,24,25] self-administered questionnaires were used to collect data on sociodemographic, lifestyle, and behavioural characteristics, including sex, age, educational level, smoking status, weight and height, and leisure-time physical activity (international physical activity questionnaire [26]).

#### 2.3.2. Dietary Data and Estimation of Dietary Polyphenols Intakes

At baseline, and every sixth-months, participants were invited to complete three non-consecutive validated [27,28,29] web-based 24-h dietary records, randomly distributed between week and weekend days to account for intra-individual variability. Participants reported all foods and beverages consumed at each meal or any other eating occasion. Portion sizes were assessed using photographs directly included in the computerised interface, from a validated picture booklet [30], if the exact quantity consumed in grams or volume was unknown by the participant. Intakes of nutrients and foods were estimated using a published food composition database [31]. We excluded energy under-reporting participants using the method proposed by Black [32].

The Phenol-Explorer database was used to estimate intake of total polyphenols, and the main classes and subclasses of polyphenols (www.phenol-explorer.eu) [16]. The total polyphenol content for a given food was computed by summing all individual polyphenols estimated as determined by chromatography, except for proanthocyanidins, for which content data obtained by normal phase HPLC were used [33], and for total intake of polyphenols quantified by the Folin Ciocalteu assay [16], or by summing all individual polyphenols. The effect of food cooking and processing was also accounted for by applying polyphenol-specific retention factors from Phenol-Explorer [34] to foods when needed to improve the reliability of the intakes estimation. This was done by creating a table containing polyphenol contents for each food item, and then multiplying the average retention factor of each cooked item with the quantity of each polyphenol (polyphenol classes/subclasses or individual polyphenols).

### 2.4. Statistical Analyses

Descriptive baseline characteristics are reported as mean ± SD (Standard Deviation) or percentage across total polyphenols quartiles. Reported *p*-values referred to Kruskal-Wallis rank sum test for continuous variables or chi^2^ tests for categorical variables. Sex-specific tertiles were computed for total polyphenols and each class of polyphenol, and each was used in separated models. Hazard ratios (HRs) and 95% confidence intervals (CIs) obtained from Cox proportional hazards models with age as the primary time variable were used to estimate the association between intakes of polyphenols and CVDs risk. Participants contributed person-time to the Cox models until the date of cardiovascular event, the date of the last completed questionnaire, the date of death, or 31 June 2017, whichever occurred first. We controlled for age (time-scale); sex; number of dietary records; BMI (Body Mass Index) (kg/m^2^); physical activity (missing/low/moderate/low), computed following IPAQ (International Physical Activity Questionnaire) recommendations [26]; smoking status (non/former/current smokers); educational level (<high-school degree/≥high-school degree); family history of CVD (yes/no); energy intake without alcohol (kcal/day); alcohol intake (g/day); and season of completion of baseline 24-h dietary records (spring/summer or fall/winter). In sensitivity analyses, we also tested the additional adjustment for baseline hypertension, type 2 diabetes, dyslipidaemia, medical treatment for these conditions, folates, and fibers. Non-linear associations between intakes of either total polyphenol or each subclass of polyphenol and cardiovascular disease risk were tested using restricted cubic spline (RCS) functions using the SAS^®^ macro written by Desquilbet and Mariotti [35]. There was no evidence of non-linearity for the association between the intakes of anthocyanins, catechins, and flavonols, and the risk of incident CVDs (RCS test, for non-linearity, *p* = 0.25, *p* = 0.19, *p* = 0.33, respectively). Therefore, polyphenol intakes were coded as a continuous variable and as sex-specific tertiles. Because of their high number (>30%), non-consumers of dihydroflavonols, theaflavins, and isoflavonoids constituted the first group, and second to third groups were coded as the sex specific median of consumers. 

All *p* values were 2-sided. For all covariates except physical activity, less than 5% of values were missing and were replaced by the mode. For physical activity (14.0% missing values), a “missing class” was introduced into the models. We defined subgroup analyses a priori to examine effect modification by the type of CVDs (CHD or CD). SAS^®^ version 9.3 (SAS^®^ Institute, Cary, 133 NC, USA) was used for analyses.

## 3. Results

### 3.1. Description of the Study Population

From the 96,716 participants to the NutriNet-Santé study who had provided at least three valid 24 h dietary records during the first two years of follow-up, 12,558 were excluded because they had a CVD or cancer before baseline or had a follow-up length under two years. The remaining 84,158 participants were thus included in the present analyses (Figure S1). 

Overall, 602 first incident major cardiovascular events (309 CHD and 293 CD) were recorded during a median of 4.9 ± 1.6 years of follow-up. Mean number of dietary records was 6.6 ± 2.8 per subject. Among subjects who experienced a cardiovascular event, the mean age at cardiovascular event was 58.3 ± 11.5 years.

Mean total intake of polyphenols (sum of individual polyphenols) was 999 ± 484 mg/day: 975 ± 478 mg/day for women and 1087 ± 498 for men (Supplementary Materials Table S1). Table 1 shows the main foods contributing to the intake of polyphenol classes and subclasses. We observed that coffee (49%), tea (23%), fruits (17%), vegetables (8%), and wine (5%) were the main contributors to total polyphenol intake. We also observed that strawberries and cherries were the main contributors to anthocyanin intake.

Compared to participants with higher total polyphenol intakes, those with lower intakes tended to be younger, non-smokers, less physically active, had lower alcohol, energy intakes, and BMI, were more likely to have a family history of CVD, and had a lower education level (all *p* values < 0.001, Table 2) than those with higher dietary polyphenol intakes.

### 3.2. Intake of Polyphenols and CVD Risk

After multivariable adjustment, we observed a 34%, 26%, and 25% reduction in the risk of major cardiovascular events compared participants in the highest vs. the lowest tertiles of anthocyanins, catechins, and flavonols, respectively (anthocyanins: HR_for a 1-point increment of 10 mg/day_ = 0.98 (0.96–0.99, *p* = 0.03, HR_T3vs.T1_ = 0.66 (0.52–0.83), *p*_trend_ = 0.0003; catechins: HR_for a 1-point increment of 10 mg/day_ = 0.98 (0.96–0.99), *p* = 0.02, HR_T3vs.T1_ = 0.74 (0.60–0.91), *p*_trend_ = 0.004; flavonols: HR_for a 1-point increment of 10 mg/day_ = 0.94 (0.90–0.99), *p* = 0.02, HR_T3vs.T1_ = 0.75 (0.61–0.94), *p*_trend_ = 0.006, Table 3).

After multivariable adjustment, comparing participants in the highest vs. the lowest tertile, the intakes of dihydrochalcones, dihydroflavonols, proanthocyanidins, hydroxybenzoic acids, stilbenes, and total polyphenols (Folin-Ciocalteu assay, but not sum of total individual polyphenols) were associated with a decrease (13%, 19%, 24%, 24%, and 27%, respectively) in the risk of total CVDs (Table 3).

### 3.3. Stratification by the Type of CVDs

After stratification by the type of CVDs, the intakes of anthocyanins and catechins were associated with both a decreased risk of CHD and CD. Dietary intakes of flavonols and stilbenes were associated only with a decreased risk of CD (Table 3).

### 3.4. Sensitivity Analyses

Sensitivity analyses provided similar results after adjustment for baseline self-reported hypertension, type 2 diabetes, or dyslipidaemia and corresponding medication, as well as intake of fibers and folates (except for chalchones and dihydroflavonols, Table S2).

## 4. Discussion

In this large prospective study, three categories of polyphenols showed strong linear associations with a decreased risk of CVDs: Anthocyanins, catechins, and flavonols. Dihydrochalcones, dihydroflavonols, hydroxybenzoic acids, stilbenes, proanthocyanidins, and total polyphenols were also related to a decreased risk of CVDs (19 to 27%).

To our knowledge, few prospective studies on the association between polyphenol classes and subclasses and CVD risk were based on such detailed polyphenol intake data considering coefficient cooking factors as well as a broad range of classes of polyphenols. Most previous studies only focused on flavonoids. Consistent with our findings, studies evaluating the associations between flavonoids and the risk of CVDs have shown significant inverse associations [36,37]. In particular, the results of a meta-analysis published by Wang et al. reported an inverse association between flavonoids and the risk of CVDs [36]. This result was also reported in a recent meta-analysis between dietary flavonoids and all-cause mortality [38]. In particular, one of the strong linear associations observed in our study was the inverse relationship between catechin intake and the risk of CVDs. We have also observed significant inverse associations for the flavonoids, ‘dihydrochalcones’ and ‘dihydroflavonols’, even though few studies have investigated these categories, since these categories are rarely quantified and only a few milligrams are consumed per day. Very few studies have shown an inverse linear relationship of anthocyanin intake with the risk of CVDs, except for investigations of data from the PREDIMED study and from the Nurses’ Health Study [18,39]. Flavanols have only been shown to be inversely associated with the risk of CVD in a single study [40], since this category has often been grouped with other flavonoids [21,41,42,43,44,45]. To our knowledge, the only study that showed a link between the risk of CVD and proanthocyanidins has focused on myocardial infarction mortality, using a US polyphenol composition table [44]. 

In the present study, we have also observed inverse associations for stilbenes, hydroxybenzoic acids, and total intake of polyphenols with the risk of CVD, although RCS procedure revealed a non-linear shape. Few other studies have been able to study these relationships in observational cohorts, since composition data for these classes of polyphenols have only been available recently in the Phenol-Explorer database [46]. Stilbenes and hydroxybenzoic acids were inversely associated with CVD risk, mostly in one study, a cohort of participants over 55 years of age of the PREDIMED study [17,18].

Regarding the total intake of polyphenols, only the PREDIMED study evaluated the total intake of polyphenols and the prospective risk of CVD, and showed, concordantly with our study, an inverse association between total polyphenol intake, assessed as the sum of individual polyphenols via the Phenol-Explorer database, and the risk of CVD and all-cause mortality [17,18]. Intriguingly, in contrast to the PREDIMED study, which used the sum of individual polyphenols as an indicator, we only found a significant result when estimating total polyphenol intakes on the basis of the Folin-Ciocalteu method. This spectrophotometric method is the most widely used experimental method to quantify total polyphenols, but is less specific since it includes antioxidant compounds other than polyphenols and better reflects the total antioxidant activity in foods [47]. Several studies showed that higher total antioxidant activity improves endothelial function [48], and reduces systemic inflammation [49]. In our study, it is possible that total antioxidant activity included other constituents, such as ascorbic acid [50], that may have had beneficial or synergetic effects on CVDs, in contrast to analyses in which we specifically focused on polyphenols. This may explain the divergence between our findings concerning total antioxidant activity and those concerning total and specific polyphenols.

In turn, no significant result concerning flavones, flavanones, isoflavonoids, hydroxycinnamic acids, and lignans were observed in our study. This is consistent with the fact that very few studies have found significant results for these specific polyphenol groups [17,18,19,41,51,52,53,54], except for flavanones [19,41,51,53], mainly present in citrus and orange and derivative juices. In addition to a possible lower consumption of these products in our cohort, various methodological differences between our study and those investigations that have obtained significant findings for flavanones could explain these inconsistencies. Indeed, significant findings for these polyphenols have been observed in studies that used the USDA’s US composition table [55], which could provide more comprehensive estimates of isoflavonoids intake [56], that estimated dietary intakes on the basis of food frequency questionnaires [56], and the investigated outcomes differed (stroke and CVD mortality) from those that the present study focused on [41,44]. In addition, one study focused on a specific sample composed by menopausal women [51], and only one study using the Phenol-Explorer^®^ table and the USDA table showed an inverse association between flavanones and all-cause mortality in a Spanish cohort [19]. In the EPIC cohort, lignans were not associated with CVD mortality risk [20], while in the PREDIMED study, lignans were inversely associated with both CVD risk and CVD mortality risk [19]. Thus, a possible reason for the lack of association observed in our cohort, and the contrasted results observed in the literature, could be the limited consumption of lignans main dietary sources in our study (oilseed-rich foods) [57], as compared to the Spanish cohort from PREDIMED [19,58]. To our knowledge, only the PREDIMED study has evaluated phenolic acids in relation to CVDs, and similarly to our study, found no significant association for hydroxycinnamic acids (for which, the main contributor is coffee), but a significant inverse association between hydroxybenzoic acids (for which, the main contributor is tea) and the risk of CVD [19]. 

Analyses stratified by the type of cardiovascular events in our study, revealed that the associations observed for stilbenes and flavonols were only found for CD, whereas the associations observed for anthocyanins and catechins were found for both CHD and CD, suggesting a particularly important role of anthocyanins and catechins for the prevention of CVD. In addition, our findings indicate that for stilbenes and flavonols, there may be a pathophysiological mechanism that is specifically related to the aetiology of CD. Flavonols have already been suggested to be inversely associated with CD risk by Hollman et al. [59,60]. Concerning stilbenes, resveratrol is probably the most well-known and studied polyphenol in the context of CVDs, and could explain the potential, but controversial, protective role of red wine consumption that has been observed concerning cerebrovascular events [61,62,63,64]. A diet rich in fruits and vegetables and, to a lesser extent, tea (the main contributor to flavonols intake), and probably/controversially a diet containing red wine (the main contributor of stilbenes) could prevent CD—which is in line with findings concerning a protective role of the Mediterranean diet [65].

Strengths of our study include its prospective design and large sample size, detailed data on risk factors and confounders related to CVDs risk, the precise evaluation of polyphenol intakes using validated 24-h dietary records coupled with the most complete and detailed polyphenol database, Phenol-Explorer database, as well as the validation of cardiovascular events by a medical committee. Generalization of our findings to populations other than French adults may be limited due to the mode of consumption and the different diets of the countries. For example, in our study, cherries and strawberries were the main contributors to anthocyanin intake while in Nordic countries, anthocyanins intake is generally provided by other berries and red fruit juices. Another limitation of our study was the limited statistical power to stratify our results on the type of CVDs. Furthermore, the multiple testing issues and the number of polyphenols have limited our ability to test each potential interaction separately. Finally, the hypothesis of residual confounding resulting from unmeasured behavioural factors and/or imprecision in the measure of included covariates cannot be entirely excluded owing to the observational design of this study. However, given our detailed adjustment for a comprehensive set of confounders, it is unlikely that these unmeasured factors would account fully for the observed results. It is possible that our findings might have been caused by other constituents found in the foods that contribute most to the classes or subclasses of polyphenols. However, the addition of other constituents of fruits, like fibers and folates, to our multivariable model did not substantially attenuate the observed associations, suggesting that polyphenols may have a protective effect against CVDs.

In conclusion, this study showed that high intakes of anthocyanins, dihydrochalcones, dihydroflavonols, catechins, proanthocyanidins, flavonols, hydroxybenzoic acids, and stilbenes, and total polyphenols were associated with a decreased risk of CVD. These subgroups of polyphenols may hence be of particular interest in the primary prevention of CVDs. Overall, our findings may have important public health implications, but will need to be confirmed in long-term randomized controlled trials, including biomarkers of CVDs risk, to elucidate mechanisms.

## Figures and Tables

**Table 1:** Contributions ^1^ of different food groups to the intake of polyphenol subclasses in the 84,158 participants of the Nutrinet Santé cohort.

Polyphenol Subclasses	Mean ± Standard Deviation	Main Food Contributors^2^ to the Intake of Subclasses of Polyphenols
**Anthocyanins**	41.8 ± 51.9	Cherries (30%), Strawberries (20%), Red wine (16%), other fruits (13%), Jam and fruit pies (5%)
**Dihydrochalcones**	2.7 ± 3.4	Apples (95%) and Apple products (5%)
**Dihydroflavonols**	2.1 ± 3.7	Red wine (86%), Grapes (7%), White wine (3%), Rosé wine (3%)
**Flavanones**	30.3 ± 31.6	Orange juice (60%), Oranges (13%), other citrus and citrus juices (11%), Red wine (2%), Tomatoes (1%)
**Flavones**	25.6 ± 13.5	Bread (43%), Oranges (20%), Wheat products (12%) Orange juices (5%)
**Flavonols**	66.6 ± 40.4	Tea (26%), Other fruits and vegetables (25%), Onions (15%), Spinach (10%), Red wine (3%)
**Isoflavonoids**	7.6 ± 26.3	Soy beverages (37%), Soy desserts (25%), Soy yoghurt (23%), Soybeans (4%)
**Hydroxybenzoic acids**	55.8 ± 75.2	Tea (48%), Chestnut and chestnut products (15%), Chicories (13%), Red wine (6%), Raspberries (5%)
**Hydroxycinnamic acids**	534 ± 418	Coffee (70%), Other fruits and vegetables (7%), Potatoes (3%), Apples (3%)
**Stilbenes**	1.5 ± 2.4	Wine (95%), Grape (3%), Strawberries (2%)
**Lignans**	1.8 ± 3.1	Multicereal bread (75%), flaxseeds (11%), Olive oil (6%)
**Catechins**	128 ± 145	Tea (75%), Herbal teas (5%), Chocolate and derivatives (3%), Apples (3%), Red wine (2%)
**Theaflavins**	17.8 ± 27.8	Tea (100%)
**Proanthocyanidins**	52.9 ± 39.5	Tea (35%), Red wine (22%), Apples and apple juices (22%), Chocolate and derivatives (14%)
**Total polyphenols (sum of individual polyphenols)**	999.3 ± 484.4	Coffee (49%), Tea (23%), Fruits (17%), Vegetables (8%), Wine (5%)
**Total polyphenols (Folin assay)**	2083.9 ± 989.9	Coffee (25%), Fruits (22%), Tea (14%), Lentils (9%), Chocolate (3%), Wine (2%)

^1^ % of total intake within each polyphenol class or subclass. Contributions were calculated by first creating a table containing polyphenol contents for each specific food item, and then computing the proportion of each food group in relation to the total amount of each specific polyphenol (contributed by all food items combined). ^2^ The first five main food contributors in each polyphenols classes or subclasses are given; a lower number of foods indicates the absence or the marginal contribution of other food contributors that contained polyphenols in the subclass considered.

**Table 2:** Baseline characteristics ^1^ of the study population overall and according to tertiles of dietary intake of total polyphenols, NutriNet-Santé Cohort, France, 2009–2017.

			Sex-Specific Tertiles ^4^ of Total Polyphenol						
	All		T1		T2		T3		*p* ^2^
**N**	84,158		28,052		28,053		28,053		
**Age (years)**	44.1	(14.5)	37.1	(14.4)	45.9	(14.1)	49.3	(12.8)	<0.0001
**Sex**									1.00
Men	17,931	(21.3)	5977	(21.3)	5977	(21.3)	5977	(21.3)	
Women	66,227	(78.7)	22,075	(78.7)	22,076	(78.7)	22,076	(78.7)	
**Educational level**									<0.0001
<high-school degree	29,848	(35.5)	11,078	(39.5)	9811	(35.0)	8959	(31.9)	
≥high-school degree	54,310	(64.5)	16,974	(60.5)	18,242	(65.0)	19,094	(68.1)	
**Smoking status**									<0.0001
Non smokers	42,572	(50.6)	16,079	(57.3)	13,935	(49.7)	9012	(32.1)	
Former smokers	28,533	(33.9)	7522	(26.8)	9904	(35.3)	10,642	(37.9)	
Current smokers	13,053	(15.5)	4451	(15.9)	4214	(15.0)	4936	(17.6)	
**Categories of BMI ^3^ (kg/m^2^)**									<0.0001
<25 kg/m^2^	59,395	(70.6)	19,498	(69.5)	19,695	(70.2)	20,202	(72.0)	
≥25 to <30 kg/m^2^	17,594	(20.9)	5816	(20.7)	6069	(21.6)	5709	(20.4)	
≥30 kg/m^2^	7169	(8.52)	2738	(9.76)	2289	(8.16)	2142	(7.64)	
**Family history of cardiovascular disease (yes)**									<0.0001
No	66,234	(78.7)	23,317	(83.2)	21,769	(77.6)	21,148	(75.4)	
Yes	17,924	(21.3)	4735	(16.9)	6284	(22.4)	6905	(24.6)	
Energy without alcohol (Kcal/day)	1869	(467)	1731	(431)	1870	(439)	2007	(495)	<0.0001
Alcohol (g/day)	7.89	(11.4)	5.49	(9.51)	8.26	(11.0)	9.91	(13.1)	<0.0001
**Physical activity ^5^**									<0.0001
High	23,847	(28.3)	6802	(24.3)	8033	(28.6)	9012	(32.1)	
Moderate	31,152	(37.0)	10,005	(35.7)	10,505	(37.5)	10,642	(37.9)	
Low	17,145	(20.4)	6563	(23.4)	5646	(20.1)	4936	(17.6)	
Missing data	12,014	(14.3)	4682	(16.7)	3869	(13.8)	3463	(12.3)	
**Number of 24 h dietary records**	6.61	(2.85)	6.13	(2.77)	6.80	(2.83)	6.90	(2.87)	<0.0001

^1^ Values are mean (± Standard deviation) for quantitative variables and frequencies N (%) for qualitative variables. ^2^ *p*-Value for the comparison between tertiles of total polyphenol intake (sum of individual polyphenols), chi-squared for categorical variables or Kruskal–Wallis rank sum test for continuous variables. ^3^ BMI: Body Mass Index = Weight (kg)/height (m)^2^. ^4^ Sex-specific cut-offs for tertiles of total intake of polyphenols (sum of individual polyphenols) were 745/1114 for women and 850/1239 for men. ^5^ Computed following IPAQ (International Physical Activity Questionnaire) recommendations.

**Table 3:** Multivariable ^1^ associations (hazard ratios (HR) and 95% confidence intervals (95% CI)) between continuous or sex-specific tertiles ^2^ of polyphenol intakes and cardiovascular disease risk (total, CHD, and stroke), NutriNet-Santé Cohort, France, 2009–2017.

Categories of Polyphenols			Total CVD				CHD				Strokes	
Tertile	Cases/Non Cases	HR	CI 95%	*p*-Value * p-Trend	Cases/Non Cases	HR	CI 95%	*p*-Value * p-Trend	Cases/Non Cases	HR	CI 95%	*p*-Value * p-Trend
**Anthocyanins**		0.98	(0.96–0.99)	0.03 *		0.97	(0.94–0.99)	0.04 *		0.99	(0.96–1.01)	0.30 *
T1	150/27,902			**0.0003**	75/27,977			**0.03**	75/27,977			**0.003**
T2	215/27,838	0.83	(0.67–1.03)		110/27,943	0.86	(0.64–1.17)		105/27,948	0.80	(0.59–0.80)	
T3	237/27,816	0.66	(0.52–0.83)		124/27,929	0.71	(0.51–0.98)		113/27,940	0.61	(0.44–0.61)	
**Dihydrochalcones**		0.81	(0.63–1.04)	0.11 *		0.74	(0.50–1.02)	0.08 *		0.93	(0.65–1.32)	0.68 *
T1	180/27,872			**0.03**	96/27,956			0.06	84/27,968			0.29
T2	224/27,829	0.98	(0.80–1.20)		116/27,937	0.98	(0.74–1.29)		108/27,945	0.98	(0.73–0.98)	
T3	198/27,855	0.801	(0.65–0.99)		97/27,956	0.75	(0.56–1.02)		101/27,952	0.86	(0.63–0.86)	
**Dihydroflavonols**		0.87	(0.68–1.12)	0.30 *		0.89	(0.65–1.22)	0.52 *		0.84	(0.57–1.25)	0.41 *
T1	136/27,917			**0.04**	67/27,986			0.27	69/27,984			0.07
T2	200/27,852	0.84	(0.67–1.06)		99/27,953	0.82	(0.60–1.13)		101/27,951	0.86	(0.63–0.86)	
T3	266/27,787	0.76	(0.59–0.98)		143/27,910	0.81	(0.57–1.15)		123/27,930	0.72	(0.50–0.72)	
**Catechins**		0.98	(0.96–0.99)	0.02 *		0.97	(0.94–0.99)	0.007 *		1.00	(0.96–1.01)	0.45 *
T1	192/27,860			**0.004**	99/27,953			0.12	93/27,959			**0.01**
T2	217/27,836	0.91	(0.74–1.11)		108/27,945	0.88	(0.66–1.17)		109/27,944	0.93	(0.70–0.93)	
T3	193/27,860	0.74	(0.60–0.91)		102/27,951	0.79	(0.59–1.05)		91/27,962	0.66	(0.51–0.69)	
**Theaflavins**		0.98	(0.94–1.00)	0.16 *		0.96	(0.91–1.01)	0.15 *		0.99	(0.95–1.03)	0.54 *
T1	258/32,924			0.22	143/33,039			0.36	115/33,067			0.40
T2	141/22,774	0.92	(0.74–1.14)		63/22,852	0.89	(0.65–1.21)		78/22,837	0.953	(0.71–0.95)	
T3	203/27,858	0.89	(0.74–1.07)		103/27,958	0.89	(0.69–1.15)		100/27,961	0.889	(0.68–0.89)	
**Proanthocyanidins**		0.97	(0.95–0.99)	0.18 *		0.96	(0.93–0.99)	0.03 *		0.98	(0.95–1.02)	0.29 *
T1	166/27,886			**0.04**	84/27,968			0.14	82/27,970			0.28
T2	211/27,842	1.02	(0.83–1.25)		112/27,941	1.10	(0.82–1.46)		99/27,954	0.98	(0.72–0.98)	
T3	225/27,828	0.81	(0.65–1.00)		113/27,940	0.80	(0.58–1.10)		112/27,941	0.85	(0.62–0.85)	
**Flavanones**		1.00	(0.97–1.03)	0.87 *		1.01	(0.97–1.05)	0.62 *		0.98	(0.94–1.03)	0.43 *
T1	206/27,846			0.89	101/27,951			0.20	105/27,947			0.25
T2	228/27,825	1.06	(0.88–1.28)		113/27,940	1.10	(0.84–1.44)		115/27,938	1.02	(0.78–1.02)	
T3	168/27,885	1.01	(0.82–1.24)		95/27,958	1.21	(0.91–1.60)		73/27,980	0.83	(0.61–0.83)	
**Flavones**		1.03	(0.97–1.09)	0.36 *		1.03	(0.95–1.11)	0.46 *		1.03	(0.94–1.12)	0.57 *
T1	192/27,860			0.29	103/27,949			0.44	89/27,963			0.48
T2	213/27,840	1.138	(0.93–1.39)		104/27,949	1.048	(0.79–1.39)		109/27,944	1.24	(0.93–1.24)	
T3	197/27,856	1.124	(0.90–1.10)		102/27,951	1.128	(0.83–1.53)		95/27,958	1.12	(0.82–1.12)	
**Flavonols**		0.94	(0.90–0.99)	0.02 *		0.97	(0.94–1.01)	0.15 *		0.97	(0.94–1.00)	0.05 *
T1	165/27,887			**0.006**	85/27,967			0.22	80/27,972			**0.008**
T2	232/27,821	0.971	(0.79–1.19)		112/27,941	0.919	(0.69–1.23)		120/27,933	1.02	(0.76–1.02)	
T3	205/27,848	0.753	(0.61–0.94)		112/27,941	0.831	(0.62–1.12)		93/27,960	0.68	(0.49–0.68)	
**Isoflavonoids**		1.00	(0.96–1.03)	0.98 *		0.97	(0.92–1.03)	0.40 *		1.02	(0.98–1.06)	0.44 *
T1	204/33,878			0.75	97/34,259			0.85	114/34,242			0.43
T2	195/24,847	0.90	(0.74–1.11)		108/21,641	1.15	(0.87–1.53)		71/21,678	0.72	(0.53–0.72)	
T3	203/24,831	0.96	(0.79–1.18)		104/27,949	1.04	(0.78–1.38)		108/27,945	0.89	(0.68–0.89)	
**Hydroxybenzoics acids**		1.00	(0.99–1.01)	0.80 *		0.99	(0.98–1.01)	0.64 *		1.00	(0.99–1.02)	0.94
T1	162/27,890			**0.01**	84/27,968			0.07	78/27,974			0.09
T2	212/27,841	0.85	(0.69–1.05)		110/27,943	0.84	(0.63–1.13)		102/27,951	0.86	(0.64–0.86)	
T3	228/27,825	0.76	(0.62–0.94)		115/27,938	0.76	(0.56–1.02)		113/27,940	0.77	(0.57–0.77)	
**Hydroxycinnamics acids**		1.00	(1.00–1.00)	0.40 *		1.00	(1.00–1.00)	0.48 *		1.00	(1.00–1.00)	0.64 *
T1	130/27,922			0.36	54/27,998			0.18	76/27,976			0.95
T2	223/27,830	1.07	(0.86–1.33)		127/27,926	1.42	(1.03–1.96)		96/27,957	0.80	(0.59–0.80)	
T3	249/27,804	1.11	(0.89–1.38)		128/27,925	1.32	(0.95–1.83)		121/27,932	0.95	(0.71–0.95)	
**Stilbenes**		0.76	(0.51–1.13)	0.19 *		0.81	(0.49–1.35)	0.47 *		0.68	(0.36–1.28)	0.24 *
T1	140/27,912			**0.02**	67/27,985			0.34	73/27,979			**0.01**
T2	200/27,853	0.85	(0.68–1.06)		99/27,954	0.86	(0.63–1.19)		101/27,952	0.83	(0.61–0.83)	
T3	262/27,791	0.73	(0.57–0.94)		143/27,910	0.84	(0.59–1.19)		119/27,934	0.64	(0.45–0.64)	
**Lignans**		0.80	(0.60–1.08)	0.17 *		0.80	(0.53–1.20)	0.33 *		0.80	(0.52–1.23)	0.32 *
T1	202/27,850			0.14	111/27,941			0.06	91/27,961			0.93
T2	172/27,881	0.73	(0.59–0.90)		91/27,962	0.71	(0.53–0.95)		81/27,972	0.75	(0.55–0.75)	
T3	228/27,825	0.84	(0.69–1.03)		107/27,946	0.75	(0.56–0.99)		121/27,932	0.95	(0.71–0.95)	
**Sum of total individual polyphenols**		1.00	(1.00–1.00)	0.35 *		1.00	(1.00–1.00)	0.12 *		1.00	(1.00–1.00)	0.82 *
T1	127/27,925			0.22	88/27,964			0.11	73/27,979			0.93
T2	234/27,819	0.99	(0.80–1.23)		114/27,939	1.00	(0.74–1.33)		111/27,942	1.13	(0.82–1.13)	
T3	241/27,812	0.880	(0.70–1.10)		107/27,946	0.79	(0.57–1.08)		109/27,944	0.954	(0.68–0.95)	
**(Total polyphenols)**		1.00	(1.00–1.00)	0.11 *		1.00	(1.00–1.00)	0.13 *		1.00	(1.00–1.00)	0.49 *
T1	161/27,891			**0.03**	78/27,974			0.05	68/27,984			0.30
T2	225/27,828	0.86	(0.69–1.06)		123/27,930	0.80	(0.60–1.07)		106/27,947	0.926	(0.68–0.93)	
T3	216/27,837	0.78	(0.62–0.97)		108/27,945	0.73	(0.53–0.99)		119/27,934	0.843	(0.61–0.84)	

^1^ Models were adjusted for age (time-scale), BMI (kg/m^2^, continuous), physical activity (high, moderate, low), smoking status (never smokers, former smokers, occasional smokers, smokers), numbers of dietary records (continuous), alcohol intake (g/d, quintiles), energy intake (without alcohol, g/d, continuous), family history of cardiovascular diseases (yes/no), educational level (<high-school degree/≥ high-school degree), and season of completion of 24-h dietary records (spring/summer, fall/winter). ^2^ Sex-specific cut-offs for tertiles of total intakes of polyphenols were 744.6/1113.8 for women and 849.6/1239.2 for men. * *p*-Value for the continuous intakes of polyphenols classes or subclasses.

## Supplementary Materials

The following are available online at http://www.mdpi.com/2072-6643/10/11/1587/s1, Table S1: Mean values of different subclasses of polyphenols by sex-specific tertiles of total polyphenols, and cut-points for polyphenol tertiles. Table S2: Multivariable associations (hazard ratios (HR) and 95% confidence intervals (95% CI)) between continuous or sex-specific tertiles of polyphenol intakes and cardiovascular disease risk (total, CHD and stroke), NutriNet-Santé Cohort, France, 2009-2017 with additional adjustments. Figure S1: Flow chart of the study population.
