# Supplementary material for: Prospective Association between Total and Specific Dietary Polyphenol Intakes and Cardiovascular Disease Risk in the Nutrinet-Santé French Cohort
Source: Nutrients. 2018 Oct 29;10(11):1587. doi: 10.3390/nu10111587 (PMC6266343; doi:10.3390/nu10111587)
Supplement: Supplementary file 1 [file nutrients-10-01587-s001.pdf]

Table S1. Mean values of different subclasses of polyphenols by sex-specific tertiles of total polyphenols, and cut-points for polyphenol tertiles.

	Sex-specific tertiles of total polyphenol								Cut-off values of tertiles		
	**All**		**T1**		**T2**		**T3**		**Men**	**Women**	**P[2]**
**N**	84 158		28052		28053		28053				
**Sum of total individual polyphenols**	**999**	**(484)**	**613**	**(165)**	**997**	**(116)**	**1388**	**(402)**	**850/1239**	**745/1114**	**<0.0001**
(Total polyphenols intake. Folin assay)	2084	(990)	1196	(1428)	1939	(236)	3117	(943)	1836/2601	1546/2212	<0.0001
Anthocyanins	41.8	(51.9)	27.5	(30.3)	43.10	(42.0)	55.0	(69.7)	22.8/56.3	14.1/39.3	<0.0001
Dihydrochalcones	2.70	(3.41)	1.72	(2.55)	2.62	(3.13)	3.77	(4.20)	0.92/3.50	0.77/2.68	<0.0001
Dihydroflavonols	2.05	(3.67)	1.03	(1.94)	2.10	(3.21)	3.02	(4.88)	0.00/0.76	0.00/0.83	<0.0001
Catechins	128	(145)	75.4	(64.9)	131	(114)	178	(196)	32.5/87.7	37.1/155.7	<0.0001
Theaflavins	17.8	(27.8)	10.3	(12.7)	18.5	(22.1)	24.5	(38.4)	0.00/15.6	0.00/20.7	<0.0001
Proanthocyaninidins	52.9	(39.5)	33.7	(21.9)	53.2	(31.8)	71.7	(49.7)	37.0/72.1	29.1/57.5	<0.0001
Flavanons	30.3	(31.6)	30.1	(33.1)	30.1	(30.9)	30.7	(30.9)	11.8/42.0	10.0/33.0	0.04
Flavones	25.6	(13.5)	23.3	(11.6)	26.0	(13.2)	27.6	(15.2)	23.7/38.4	18.2/26.8	<0.0001
Flavonols	66.6	(40.3)	46.5	(26.6)	67.0	(33.1)	86.3	(48.3)	47.0/75.8	44.0/75.6	<0.0001
Isoflavonoids	7.60	(26.3)	4.50	(20.9)	6.70	(25.4)	11.5	(31.3)	0.00/0.03	0.00/0.12	<0.0001
Proanthocyaninidins	261	(218)	185	(207)	252	(210)	347	(234)	152/271	178/318	<0.0001
Hydroxybenzoic acids	55.8	(75.1)	32.9	(27.3)	54.5	(46.7)	79.9	(110)	17.0/51.2	18.9/63.5	<0.0001
Hydroxycinnamics acids	534	(418)	301	(141)	528	(232)	773	(482)	367/738	246/614	<0.0001
Stilbenes	1.46	(2.37)	0.77	(1.27)	1.50	(2.08)	2.12	(3.13)	0.60/2.97	0.15/0.93	<0.0001
Lignans	1.77	(3.02)	1.18	(1.97)	1.75	(2.78)	2.39	(3.90)	0.82/1.36	0.69/1.22	<0.0001

[1] Values are mean ± Standard deviation. Means values and standard deviations are all expressed in Mg/day. [2] P-value for the comparison between tertiles of total polyphenols (sum of individual polyphenols), Kruskal–Wallis rank sum test for continuous variables.

Table S2. Multivariable[1] associations (hazard ratios (HR) and 95% confidence intervals (95% CI)) between continuous or sex-specific tertiles [2] of polyphenol intakes and cardiovascular disease risk (total, CHD and stroke), NutriNet-Santé Cohort, France, 2009-2017 with additional adjustments.

	**Categories of polyphenols**			**Total CVD**				**CHD**				**Strokes**
**Tertile**	**cases/non cases**	**HR**	**CI 95%**	**P-Value* P-trend**	**cases/non cases**	**HR**	**CI 95%**	**P-Value* P-trend**	**cases/non cases**	**HR**	**CI 95%**	**P-Value* P-trend**
**Anthocyanins**		0.98	(0.96–0.99)	**0.04**		0.98	(0.95–0.99)	0.08		0.99	(0.96–1.01)	0.31
T1	150/27902			**0.0007**	75/27977			0.07	75/27977			**0.007**
T2	215/27838	0.83	(0.67–1.04)		110/27943	0.87	(0.64–1.18)		105/27948	0.80	(0.59–1.09)	
T3	237/27816	0.67	(0.53–0.85)		124/27929	0.74	(0.53–1.03)		113/27940	0.61	(0.43–0.85)	
**Dihydrochalcones**		0.87	(0.66–1.13)	0.29		0.83	(0.57–1.21)	0.34		0.91	(0.62–1.33)	0.63
T1	180/27872			0.06	96/27956			0.20	84/27968			0.26
T2	224/27829	0.98	(0.80–1.20)		116/27937	0.99	(0.75–1.32)		108/27945	0.96	(0.71–1.29)	
T3	198/27855	0.82	(0.66–1.03)		97/27956	0.82	(0.60–1.12)		101/27952	0.84	(0.61–1.15)	
**Dihydroflavonols**		0.87	(0.66–1.13)	0.29		0.83	(0.57–1.21)	0.34		0.91	(0.62–1.33)	0.63
T1	136/27917			0.07	67/27986			0.22	69/27984			0.09
T2	200/27852	0.97	(0.74–1.27)		99/27953	0.81	(0.59–1.12)		101/27951	0.88	(0.64–1.21)	
T3	266/27787	0.83	(0.62–1.10)		143/27910	0.79	(0.55–1.13)		123/27930	0.73	(0.51–1.05)	

**Catechins**		0.99	(0.99–0.99)	**0.03**		0.99	(0.98–0.99)	**0.02**		0.99	(0.99–1.01)	0.23
T1	192/27860			**0.01**	99/27953			0.26	93/27959			**0.01**
T2	217/27836	0.93	(0.76–1.14)		108/27945	0.93	(0.70–1.25)		109/27944	0.93	(0.70–1.24)	
T3	193/27860	0.76	(0.62–0.94)		102/27951	0.85	(0.63–1.14)		91/27962	0.69	(0.51–0.93)	
**Theaflavins**		0.99	(0.95–1.03)	0.64		0.97	(0.92–1.02)	0.19		0.99	(0.95–1.03)	0.59
T1	258/32924			0.41	143/33039			0.44	115/33067			0.44
T2	141/22774	0.90	(0.68–1.20)		63/22852	0.90	(0.66–1.23)		78/22837	0.95	(0.71–1.29)	
T3	203/27858	0.89	(0.67–1.18)		103/27958	0.90	(0.70–1.17)		100/27961	0.90	(0.68–1.18)	
**Proanthocyanidins**		0.98	(0.96–0.99)	0.14		0.97	(0.94–0.99)	0.09		0.98	(0.95–1.02)	0.32
T1	166/27886			0.05	84/27968			0.26	82/27970			0.32
T2	211/27842	1.06	(0.86–1.30)		112/27941	1.12	(0.83–1.49)		99/27954	0.99	(0.74–1.35)	
T3	225/27828	0.85	(0.68–1.01)		113/27940	0.84	(0.61–1.17)		112/27941	0.86	(0.62–1.18)	
**Flavanones**		0.99	(0.97–1.03)	0.79		1.01	(0.97–1.05)	0.68		0.98	(0.94–1.03)	0.40
T1	206/27846			0.93	101/27951			0.21	105/27947			0.25
T2	228/27825	1.08	(0.89–1.30)		113/27940	1.12	(0.86–1.48)		115/27938	1.02	(0.78–1.34)	
T3	168/27885	0.99	(0.81–1.24)		95/27958	1.20	(0.90–1.62)		73/27980	0.82	(0.60–1.12)	

**Flavones**		1.03	(0.97–1.10)	0.31		1.04	(0.96–1.14)	0.34		1.02	(0.93–1.12)	0.65
T1	192/27860			0.32	103/27949			0.39	89/27963			0.61
T2	213/27840	1.11	(0.91–1.36)		104/27949	1.01	(0.76–1.34)		109/27944	1.22	(0.91–1.63)	
T3	197/27856	1.12	(0.90–1.40)		102/27951	1.15	(0.84–1.56)		95/27958	1.09	(0.79–1.50)	
**Flavonols**		0.98	(0.95–0.99)	**0.04**		0.99	(0.96–1.03)	0.59		0.96	(0.93–0.99)	**0.03**
T1	165/27887			**0.02**	85/27967			0.65	80/27972			**0.01**
T2	232/27821	0.99	(0.81–1.23)		112/27941	0.98	(0.73–1.32)		120/27933	1.01	(0.75–1.36)	
T3	205/27848	0.77	(0.61–0.98)		112/27941	0.93	(0.68–1.28)		93/27960	0.64	(0.45–0.89)	
**Isoflavonoids**		0.98	(0.95–0.99)	0.06		0.99	(0.96–1.03)	0.59		0.96	(0.93–0.99)	**0.03**
T1	204/33878			0.89	97/34259			0.45	114/34242			0.48
T2	195/24847	0.93	(0.76–1.15)		108/21641	1.14	(0.86–1.52)		71/21678	0.73	(0.54–0.99)	
T3	203/24831	0.98	(0.80–1.20)		104/27949	1.12	(0.84–1.50)		108/27945	0.90	(0.68–1.19)	
**Hydroxybenzoics acids**		0.99	(0.99–1.01)	0.76		0.99	(0.98–1.02)	0.79		0.99	(0.99–1.02)	0.88
T1	162/27890			**0.04**	84/27968			0.19	78/27974			0.11
T2	212/27841	0.88	(0.71–1.09)		110/27943	0.88	(0.66–1.19)		102/27951	0.88	(0.64–1.19)	
T3	228/27825	0.79	(0.64–0.99)		115/27938	0.81	(0.60–1.10)		113/27940	0.77	(0.56–1.06)	

**Hydroxycinnamics acids**		0.99	(0.99–0.99)	0.33		0.99	(0.99–0.99)	0.34		0.99	(0.99–0.99)	0.68
T1	130/27922			0.34	54/27998			0.13	76/27976			0.87
T2	223/27830	1.06	(0.85–1.32)		127/27926	1.45	(1.04–2.01)		96/27957	0.78	(0.57–1.06)	
T3	249/27804	1.11	(0.89–1.39)		128/27925	1.36	(0.98–1.90)		121/27932	0.93	(0.69–1.26)	
**Stilbenes**		0.79	(0.53–1.18)	0.25		0.87	(0.52–1.45)	0.58		0.70	(0.37–1.31)	0.26
T1	140/27912			**0.01**	67/27985			0.29	73/27979			**0.01**
T2	200/27853	0.84	(0.67–1.05)		99/27954	0.86	(0.62–1.19)		101/27952	0.82	(0.60–1.12)	
T3	262/27791	0.72	(0.56–0.93)		143/27910	0.82	(0.57–1.17)		119/27934	0.64	(0.45–0.91)	
**Lignans**		0.85	(0.63–1.15)	0.28		0.89	(0.59–1.34)	0.58		0.80	(0.52–1.25)	0.33
T1	202/27850			0.29	111/27941			0.16	91/27961			0.97
T2	172/27881	0.73	(0.59–0.90)		91/27962	0.72	(0.54–0.96)		81/27972	0.75	(0.55–1.02)	
T3	228/27825	0.87	(0.71–1.07)		107/27946	0.80	(0.60–1.07)		121/27932	0.96	(0.71–1.29)	
**Sum of total individual polyphenols**		0.99	(0.99–0.99)	0.39		0.99	(0.99–0.99)	0.67		0.99	(0.99–0.99)	0.44
				0.12				0.29				0.29
T1	127/27925	0.89	(0.71–1.10)		88/27964	0.86	(0.64–1.15)		73/27979	0.93	(0.68–1.27)	
T2	234/27819	0.82	(0.64–1.05)		114/27939	0.83	(0.59–1.17)		111/27942	0.83	(0.58–1.19)	

T3	241/27812	0.99	(0.99–0.99)	0.71	107/27946	0.99	(0.99–0.99)	0.83	109/27944	0.99	(0.99–0.99)	0.74
**(Total polyphenols)**				0.69				0.42				0.57
T1	161/27891	1.05	(0.84–1.31)	**0.04**	78/27974	0.96	(0.70–1.31)	0.21	68/27984	1.15	(0.83–1.60)	0.23
T2	225/27828	0.97	(0.76–0.97)		123/27930	0.99	(0.71–1.38)		106/27947	0.95	(0.67–1.35)	
T3	216/27837				108/27945				119/27934			

[1] Models were adjusted for age (time-scale), BMI (kg/m^2, continuous), physical activity (high, moderate, low), smoking status (never smokers, former smokers, occasional smokers, smokers), numbers of dietary records (continuous), alcohol intake (g/d, quintiles), energy intake (without alcohol, g/d, continuous), family history of cardiovascular diseases (yes/no), educational level (<high-school degree/≥ high-school degree), season of completion of 24-h dietary records (spring/summer, fall/winter), baseline hypertension, type 2 diabetes, dyslipidaemia, medical treatment for these conditions, folates (continuous) and fibers (continuous).[2] Sex-specific cut-offs for tertiles of total intakes of polyphenols were 744.6/1113.8 for women and 849.6/1239.2 for men. * *p*-Value for the continuous intakes of polyphenols classes or subclasses.

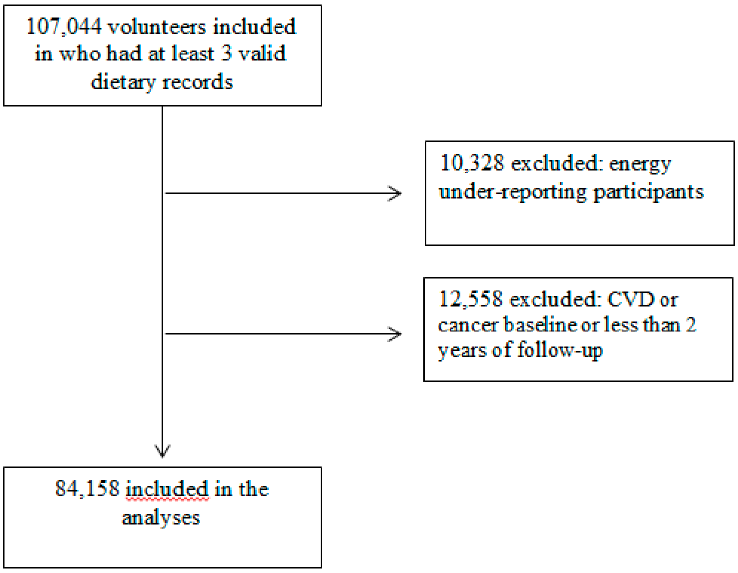

Figure S1. Flow diagram of participants included in the analyses of intakes of polyphenols and cardiovascular diseases risk.
